# Liver-specific overexpression of HKDC1 increases hepatocyte size and proliferative capacity

**DOI:** 10.1038/s41598-023-33924-3

**Published:** 2023-05-17

**Authors:** Carolina M. Pusec, Vladimir Ilievski, Adam De Jesus, Zeenat Farooq, Joseph L. Zapater, Nadia Sweis, Hagar Ismail, Md Wasim Khan, Hossein Ardehali, Jose Cordoba-Chacon, Brian T. Layden

**Affiliations:** 1 Division of Endocrinology, Diabetes, and Metabolism, Department of Medicine, University of Illinois at Chicago, Chicago, IL USA; 2 Northwestern University Feinberg School of Medicine, Chicago, IL USA; 3 Jesse Brown VA Medical Center, Chicago, IL USA

**Keywords:** Endocrinology, Endocrine system and metabolic diseases, Metabolism, Liver, Hepatocytes

## Abstract

A primary role of the liver is to regulate whole body glucose homeostasis. Glucokinase (GCK) is the main hexokinase (HK) expressed in hepatocytes and functions to phosphorylate the glucose that enters via GLUT transporters to become glucose-6-phosphate (G6P), which subsequently commits glucose to enter downstream anabolic and catabolic pathways. In the recent years, hexokinase domain-containing-1 (HKDC1), a novel 5th HK, has been characterized by our group and others. Its expression profile varies but has been identified to have low basal expression in normal liver but increases during states of stress including pregnancy, nonalcoholic fatty liver disease (NAFLD), and liver cancer. Here, we have developed a stable overexpression model of hepatic HKDC1 in mice to examine its effect on metabolic regulation. We found that HKDC1 overexpression, over time, causes impaired glucose homeostasis in male mice and shifts glucose metabolism towards anabolic pathways with an increase in nucleotide synthesis. Furthermore, we observed these mice to have larger liver sizes due to greater hepatocyte proliferative potential and cell size, which in part, is mediated via yes-associated protein (YAP) signaling.

## Introduction

One of the main physiologic functions of the liver is to regulate whole body glucose and energy homeostasis^1^. The liver takes up glucose largely via the GLUT2 transporter and subsequently, the enzyme glucokinase (GCK) phosphorylates it to glucose-6-phosphate (G6P), which commits glucose to enter various anabolic and catabolic pathways within the hepatocyte^2,3^. During the post-prandial state, G6P molecules proceed through glycolysis, enter the tricarboxylic acid (TCA) cycle, and provide intermediates along the way that give rise to various processes including oxidative phosphorylation. With excess energy, G6P and downstream intermediates largely contribute toward energy storage in the form of glycogen or other nutrients including triglycerides. To maintain physiologic blood glucose levels during the fasting state, hepatocytes undergo glycogenolysis followed by gluconeogenesis under extended periods of fasting^2^. During periods of stress, such as observed in nonalcoholic fatty liver disease (NAFLD), a state of excess fat stored in the liver that may contain elements of injury, inflammation, and fibrosis, the liver tries to retain functionality through its capacity to regenerate by re-entering the cell cycle despite the hepatocytes being highly differentiated^4^. This adaptive response compensates for the loss of functional tissue via hepatocyte hypertrophy and/or hyperplasia. However, with chronic damage, protective mechanisms can at times fail to prevent irreversible states such as cirrhosis or hepatocyte transformation into hepatocellular cancerous cells in the setting of un-regulated proliferation^5^.

A novel 5th hexokinase termed hexokinase domain containing-1 (HKDC1) was suggested by Irwin et al.^6^ and has been verified and characterized by our laboratory and other groups since its initial report^7–14^. Its importance was first highlighted in a genome-wide association study (GWAS) that identified genome-wide association of variants within HKDC1 and gestational glucose metabolism^7^. Subsequent studies have now shown that, in part, the action of HKDC1 is through its role in the liver during pregnancy^11^. Our findings have, thus far, demonstrated HKDC1’s enzymatic activity for converting glucose to G6P to be less than HK1-3 and glucokinase (GCK)^10^. Also, the expression profile of HKDC1 varies considerably across tissues^7,8^, and its expression in the liver is relatively low. However, the expression of hepatic HKDC1 expression is increased in NAFLD^14^ and other liver conditions^15^.

In the recent years, we began exploring hepatic HKDC1 and its impact on whole body glucose homeostasis by overexpression or deletion of HKDC1 in hepatocytes during pregnancy, where we observed that hepatic HKDC1 expression is beneficial for maintaining whole-body glucose homeostasis via its action on regulating insulin sensitivity^11^. We also tested the effects of acute (7-day) in vivo adenovirus-mediated overexpression of HKDC1 in male mice. Interestingly, HKDC1 contains an N-terminal mitochondrial-binding peptide sequence that localizes to VDAC and impacts glycolytic capacity, maximal respiration and glucose oxidation—all signs that HKDC1 affects mitochondrial function^10^. Because the expression of hepatic HKDC1 increases dramatically in NASH^14^, HCC^12^, and most recently discovered in alcoholic liver disease (ALD)^16^, we sought to determine if chronic hepatic overexpression of HKDC1 is sufficient to remodel hepatic metabolic pathways in order to understand its role in liver disease progression. To do this, we developed a chronic, stable overexpression of hepatic HKDC1 via the use of adeno-associated virus serotype 8 (AAV-8) in adult mice on a low-fat diet to delineate its role in the development of liver disease in the absence of a diet-induced liver disease model. Our data show that chronic hepatic HKDC1 overexpression impacts whole body glucose homeostasis over time, and importantly, shifts liver glucose metabolism to a more anabolic state resulting in an increase in nucleotide synthesis. The expression of HKDC1 also leads to greater liver size due to increased hepatocyte size and is, in part, due to the upregulation of YAP and its downstream effects.

## Methods

### Animal models and diets

All mice were obtained from The Jackson Laboratory and housed under a 14-h light, 10-h dark cycle with ad libitum access to normal chow (Envigo), unless otherwise noted. All mouse studies were approved by Institutional Animal Care and Use Committee and performed in accordance with the Guide for the Care and Use of Laboratory Animals at the University of Illinois at Chicago. For experiments requiring euthanasia, an intraperitoneal injection of ketamine/xylazine was administered to induce deep anesthesia followed by cervical dislocation. This method is consistent with AVMA guidelines for euthanasia. Further, the studies proposed here are reviewed by the Animal Care and Use Committee at UIC. This study is reported in accordance with ARRIVE guidelines.

Mice were injected with a single dose of adeno-associated virus serotype 8 (AAV-8) that has a hepatocyte-specific promoter thyroxin-binding globulin promoter (TBGp) via the lateral tail vein at 10 weeks of age. A single injection of 1.5*10e11 genome copies of AAV8-TBGp-full-length-humanHKDC1 induces the stable expression of human HKDC1 in hepatocytes (hHKDC1 OE) where mice injected with AAV8-TBGp served as the controls. AAV vectors were generated by Penn Vector Core, University of Pennsylvania. After injection, the mice were switched to a low-fat diet (D09100304; Research Diets, New Brunswick, NJ) for 16 weeks. Whole-body fat and lean mass were measured with a minispec LF50 Body Composition Analyzer (NMR) (Bruker, Billerica, MA) at 16 weeks after which, the mice were euthanized for end-point analyses.

### Glucose tolerance test

Intraperitoneal glucose tolerance tests (IPGTTs) were performed as previously described^14^. Briefly, mice were fasted for 16 h overnight with glucose given by IP injection at a dose of 2 g glucose/kg body weight. Glucose levels were measured from blood obtained from tail veins and measured with a One-Touch Ultra Glucometer and taken at multiple time points (from 0 to 120 min) during the IPGTT.

### Plasma glucose and insulin levels

For glucose, blood was obtained from tail veins and measured using One-Touch Ultra Glucometer after a 4-h fast. For insulin, blood was collected via the tail vein utilizing heparinized capillary tubes after which they were centrifuged at 7000 rpm for 15 min for plasma collection and insulin levels were measured using the Mouse Ultrasensitive Insulin ELISA kit (80-INSMSU-E01, ALPCO).

### Western-blot

Fresh frozen liver tissues were homogenized in lysis buffer containing 50 mM Tris–HCl (pH 7.4), 100 mM NaCl, 1 mM EDTA, 1 mM EGTA and 1% Triton X-100 with protease/phosphatase inhibitor mixture (Complete, Roche). Protein concentration was determined using Pierce™ Rapid Gold BCA Protein Assay Kit (A53227, Thermo Fisher Scientific, Waltham, MA). Equal amounts of protein were loaded on an SDS-PAGE 4–15% precast polyacrylamide gel (5671081, Bio-Rad Laboratories, Inc, Hercules, CA) and resolved by electrophoresis. Protein was transferred to a 0.45 μm nitrocellulose membrane. Membranes were blocked in 5% milk/Tris-buffered saline-Tween 20, and primary antibody was diluted 1:1000 in 5% BSA/Tris-buffered saline-Tween 20 (unless otherwise specified) while secondary antibody incubations were performed in 5% milk/Tris-buffered saline-Tween 20 at a 1:5000–1:10,000 dilution. All primary antibodies were incubated overnight at 4 °C, and secondary incubations were for at least 60 min at room temperature. Antibodies were as follows: Yes associated protein (YAP) (Proteintech Group, Inc., Rosemont, IL; catalog no. 13584-1-AP, RRID:AB_2218915), Cysteine-rich protein 61 (CYR61) (Proteintech Group, Inc.; catalog no. 26689-1-AP, RRID:AB_2880604), Connective tissue growth factor (CTGF) (Proteintech Group, Inc.; catalog no. 23936-1-AP, RRID:AB_2736836), Cadherin-associated protein, beta 1 (Beta-Catenin) (Proteintech Group, Inc; catalog no. 51067-2-AP RRID:AB_2086128), Glyceraldehyde-3-phosphate dehydrogenase (GAPDH) (1:5000) (Cell Signaling Technology, Inc., Danvers, MA; catalog no. D16H11 RRID:AB_10622025), Hexokinase Domain Containing-1 (HKDC1) (ABCAM Cambridge, MA; catalog no. ab228729)), Glucokinase (GCK) (Santa Cruz Biotechnology Inc., Dallas, TX; catalog no. sc-7908 RRID:AB_2107620), Hexokinase I (HKI) (Cell Signaling Technology, Inc., Danvers, MA; catalog no. 2024, RRID:AB_2116996), Hexokinase 2 (HK2) (Cell Signaling Technology, Inc., Danvers, MA; catalog no. 2867, RRID:AB_2232946), Hexokinase 3 (HK3) (Proteintech Group, Inc.; catalog no. 13333-1-AP), Pyruvate kinase M2 isoform (PKM2) (Cell Signaling Technology, Inc., Danvers, MA; catalog no. 4053, RRID:AB _1904096), Lamin A/C (Cell Signaling Technology, Inc., Danvers, MA; catalog no. 4777 RRID:AB_10545756), and Translocase of outer mitochondrial membrane 20 homolog (TOM20) (Proteintech Group, Inc.; catalog no. 11802-1-AP RRID:AB_2207530). Blots were imaged and analyzed using a ChemiDoc MP Imager (Bio-Rad), and Image Lab software, version 6.0 (Bio-Rad), respectively.

### Nuclear and cytoplasmic extraction

Tissue lysis and extraction of cytoplasmic and nuclear protein fractions were conducted using the Thermo Scientific NE-PER Nuclear and Cytoplasmic Extraction Kit (catalog number: 78833). Tissue preparation was performed per the manufacturer’s instructions. Briefly, 40 mg of liver tissue sample was harvested, washed with PBS, and centrifuged at 500 × g for 5 min. The supernatant was discarded and the tissue was then homogenized using a Dounce homogenizer in appropriate volume of extraction reagent.

### Mitochondrial isolation

30 mg of fresh frozen liver tissue was homogenized in homogenization buffer (10 mM TrisHCl, pH 6.7, 10 mM KCl, 0.15 mM MgCl_2_) supplemented with protease and phosphatase inhibitors 10 μl/ml and 1 mM DTT and 1 mM PMSF using a 2 mL dounce homogenizer (DWK Life Sciences, LLC, Millville, NJ). After about 30 strokes, lysate was then transferred into a 1.5 mL micro-centrifuge tube containing 50 μL of 2 M sucrose. After mixing, lysate was centrifuged at 1200xg for 10 min at 4 °C and this process was repeated beginning with adding the supernatant into a fresh micro-centrifuge tube containing 50 μL of 2 M sucrose. The supernatant (the cytosolic fraction) was transferred into a fresh micro-centrifuge tube. The pellet was re-suspended in mitochondrial buffer (10 mM TrisHCl, pH 6.7, 0.15 mM MgCl_2_, 0.25 M sucrose and supplemented with protease and phosphatase inhibitors, 1 mM DTT and 1 mM PMSF) at 3 × volume of the pellet size. The lysate was then centrifuged at 10,000xg for 10 min at 4 degrees. The supernatant was discarded and the pellet was re-suspended in fresh mitochondrial buffer (~ 40 μL) and stored at − 80 °C degrees.

### RNA isolation and qPCR

RNA was extracted from 10 mg livers using TRIzol reagent (15596026, Thermo Fisher Scientific) and chloroform for phase separation. RNA was purified using RNeasy Mini Kit (74106, Qiagen, Germantown, MD.) and treated with RNase-Free DNase (79254, Qiagen). Purified RNA (1 µg) was then reverse transcribed using qScript Reverse transcription (Quanta Biosciences) and quantified via qPCR using iTaq Universal SYBR Green Supermix (1725124 Bio-Rad Laboratories, Inc.) where final primer concentrations were 0.625 µM for each reaction, and data were analyzed using the CFX Connect Real-Time PCR Detection System (Bio-Rad Laboratories, Inc). For primer sequences, please see Table 1.

**Table 1:** Primer sequences utilized for quantitative PCR.

Gene	Species	Forward primer 5′–3′	Reverse primer 5′–3′
HKDC1	Mouse	ACACTTGGTGGCGTTTTACTT	CCGCATGTGATACAGGAACC
HKDC1	Human	TCCTGGCAAGCAGAGATACG	AGACGCTCTGAAATCTGCCC
HK1	Mouse	TGCCATGCGGCTCTCTGATG	CTTGACGGAGGCCGTTGGGTT
HK2	Mouse	TGGAGATTTCTAGGCGGTTCC	TCGCCATGTTCTGTCCCATC
HK3	Mouse	CACCATTGGGCCTTCTGGCCTGCATCCT	TGGCACGCCCTCGTGTGGGCAGACTGA
GCK	Mouse	GAGGTCGGCATGATTGTG GGC A	ACACACATGCGCCCCTCATCGCC
PCNA	Mouse	TCGTCTCACGTCTCCTTGGT	TTGGACATGCTGGTGAGGTT
ß-actin*	Mouse	GGCTGTATTCCCCTCCATCG	CCAGTTGGTAACAATGCCATGT

*HKDC1* hexokinase domain containing protein-1, *HK1* hexokinase 1, *HK2* hexokinase 2, *HK3* hexokinase 3, *GCK* glucokinase, *PCNA* proliferating cell nuclear antigen, *ß-actin* beta-actin, * housekeeping gene.

### Immunofluorescence staining

Liver pieces were fixed in 4% PFA for 24 h at room temperature. The next day, fixed livers were washed in 50% ethanol and then stored in 70% ethanol until embedding in paraffin by the UIC core. Briefly, sections were first de-paraffinized with xylene and rehydrated through a series of decreasing percentages of ethanol. Antigen retrieval was performed by microwaving sections in 1 mM ethylenediaminetetraacetic acid (EDTA), pH 8.0 or in 10 mM citrate buffer pH 6.0 for 5 min repeated 4 times. Cell and nuclear membrane permeablization was performed by incubating sections in 0.25% Tween 20 in PBS for 30 min. Tissue sections were incubated with: Yes associated protein (YAP) from Proteintech Group, Inc.; catalog no. 13584-1-AP, RRID:AB_2218915, rabbit polyclonal antibody to Proliferating cell nuclear antigen (PCNA) (Santa Cruz Biotechnology, Inc.; catalog no. SC-7907, RRID:AB_2160375) at 1:100 dilution overnight at 4 °C. Appropriate secondary antibody donkey anti-rabbit antibody conjugated with Alexa Fluor 594 (A21207, Thermo Fisher Scientific) were diluted at 1:800 for 1 h at room temperature. Nuclei were stained with ProLong Gold Antifade Mountant with DAPI (P36931, Thermo Fisher Scientific, Waltham, MA). Positive-staining cells were counted in five randomly selected fields within a defined area in the liver as visualized by IF and the mean number ± SEM of positive cells per field were tabulated per group^17^.

### Wheat germ agglutinin (WGA) staining

Liver sections or AML-12 cells were labeled with Alexa 488-conjugated wheat germ agglutinin (WGA, catalog no. W11261, Invitrogen) at 5 μg/mL diluted in PBS for 10 min at room temperature followed by two washes in PBS. Stained sections were imaged using Olympus BX53 fluorescence microscope. Cells size was calculated using ImageJ (NIH, Bethesda, MD).

### Non-esterified fatty acids (NEFAs)

Quantification of plasma NEFAs was carried out with reagents from Wako Diagnostics, as per the manufacturer’s instructions.

### Hepatic triglycerides and glycogen content

Mice were fasted for 4 h. Harvested livers were immediately flash frozen in liquid nitrogen and then stored at − 80° until analyses were performed. For triglycerides, livers were homogenized on ice using a 2 mL glass dounce homogenizer (DWK Life Sciences, LLC) and PBS. Homogenates were cleared by centrifugation at 13,000 rpm, and the supernatant was collected. Hepatic triglycerides were determined using Infinity Triglyceride Solution (TR22421 Thermo Fisher Scientific). For glycogen, flash-frozen liver samples were determined using the Glycogen Assay Kit (MAK016, Sigma-Aldrich, St. Louis, MO) as per manufacturer protocol.

### De Novo lipogenesis index (DNL)

Total lipid extraction from the livers was done via Bligh and Dyer procedure^18^. Lipids were then transmethylated with 10% boron trifluoride-methanol (Sigma-Aldrich) and DNL was measured using gas chromatography/mass spectrometry (GC/MS) as described^19^.

### Hexokinase activity

Livers of 4 h-fasted mice were assayed using the Hexokinase activity kit (MAK091, Sigma-Aldrich) according to the manufacturer's instructions.

### Metabolite assays

Quantification of Glucose-6-Phosphate and Lactate levels were done using commercially available kits (ab83426 and ab65330), as per the manufacturer’s protocols. For each assay, 20 mg samples of liver tissue were harvested and washed in cold PBS, resuspended in PBS or the recommended assay buffer, and mechanically homogenized using a Dounce homogenizer. Tissue lysates were then deproteinized with TCA using a Deproteinizing Sample Preparation Kit (ab204708) and the resulting deproteinized samples were used for subsequent assays.

### Cell culture

Alpha mouse liver 12 (AML-12) cells (CRL-2254, ATCC, Manassas, VA) obtained from ATCC were cultured in DMEM:F12 medium (11-330-032, Gibco, Billings, MT) supplemented with 10% fetal bovine serum and 1% penicillin streptomycin. Additionally, the media was supplemented with insulin, transferrin, selenium (25-800-CR, ITS ™, Corning, Glendale, AZ) as per manufacturer’s instructions. Briefly, the cells were plated in T-25 (156430, Thermo Fisher Scientific) flasks and incubated in a 37 °C humidified incubator in presence of 5% CO_2_ until they formed a monolayer and achieved the desired confluence.

### Generation of lentivirus

Lentivirus containing a copy of human HKDC1 gene was generated in HEK-293 T cells as previously described^20^. After collecting the virus containing supernatant (media) from HEK-293 T cells for three consecutive days, the media was pooled, and ultracentrifugation was performed at 36,000 rpm for 2 h (8043-30-1082, L-80XP, Beckman Coulter, Brea CA) to concentrate the virus. Next, the entire virus pellet was re-suspended in 5 ml of media and 1 ml aliquots were prepared, snap frozen and stored at − 80 °C till further use.

### Generation of stable hHKDC1 cell line

AML-12 cells were cultured to a confluency of 80% in a 6-well plate and infected with adenovirus containing a copy of full length (FL) human HKDC1 gene (hHKDC1). Briefly, 1 ml of adenovirus containing hHKDC1 was incubated at room temperature for 5 min with 1 µg/ml of polybrene (TR-1003-G, Millipore Sigma). Virus was added to the cells after removing media from the well. The cells were incubated for 3 h after which fresh F12:cDMEM was added. Following infection, the cells were selected with blasticidin (A1113902, Thermo Fisher Scientific) for 7 consecutive days (10 µg/ml) and infected cells were further propagated^21^. The expression of human HKDC1 in the stable cells was further confirmed through RT-qPCR.

### Transfection

Lipofectamine based transfection using RNAi MAX lipofectamine (13778030, Thermo Fisher Scientific) was performed as per manufacturer’s protocol for knock down of HKDC1. Both wild type and human HKDC1 (hHKDC1) containing AML-12 cells were transfected with two types of siRNA SASI_Mm01_00122693 (CCAUCAAGAGGAGAAACGA[dT][dT]) and SASI_Mm01_00122694 (CCAAGUUUCGGGUCUUGAA[dT][dT]), Millipore Sigma) against mouse and human HKDC1. For mouse Yap1 silencing, we used a cocktail of two siRNAs (00022140 and 00022142, Sigma Aldrich) in order to achieve a knockdown of ~ 80%. The sequences of the siRNAs are given below: SASI_Mm01_00022140 (GCUAGAUAAAGAAAGCUUU[dT][dT]) and (AAAGCUUUCUUUAUCUAGC[dT][dT] ) and SASI_Mm01_00022142 (GGUCAAAGAUACUUCUUAA[dT][dT]) and (UUAAGAAGUAUCUUUGACC[dT][dT]). Briefly, cells were grown to a confluency of 80% in 6-well plates. For each well of a 6-well plate, 150 µl of opti-MEM (31-985-070, Gibco) was mixed with 5 µl of siRNA (100 µM). This was added to a mixture containing 150 µl of opti-MEM (31985062, Gibco) and 9 µl RNAi MAX lipofectamine and incubated for 5 min. The mixture was then added to the wells in a dropwise manner following addition of 1.5 ml of fresh opti-MEM and cells were incubated for 6 h. Next, 1.5 ml of fresh DMEM:F12 media was added to each well and incubated overnight. After 48 h, cells were harvested for protein and RNA extraction.

### Metabolomics

Liver tissues were harvested from mice after a 4 h fast and immediately flash frozen in liquid nitrogen until harvested for metabolites using a 80% methanol & 20% ultrapure water extraction protocol. Metabolomics services were performed by the Metabolomics Core Facility at Robert H. Lurie Comprehensive Cancer Center of Northwestern University. Samples were analyzed by High Performance Liquid Chromatography and High-Resolution Mass Spectrometry and Tandem Mass Spectrometry (HPLC–MS/MS). Specifically, the system consisted of a Thermo Q-Exactive in line with an electrospray source and an Ultimate3000 (Thermo) series HPLC consisting of a binary pump, degasser, and auto-sampler outfitted with an Xbridge Amide column (Waters; dimensions of 4.6 mm × 100 mm and a 3.5 µm particle size). The mobile phase A contained 95% (vol/vol) water, 5% (vol/vol) acetonitrile, 20 mM ammonium hydroxide, 20 mM ammonium acetate, pH = 9.0; B was 100% Acetonitrile. The gradient was as follows: 0 min, 15% A; 2.5 min, 30% A; 7 min, 43% A; 16 min, 62% A; 16.1–18 min, 75% A; 18–25 min, 15% A with a flow rate of 400 μL/min. The capillary of the ESI source was set to 275 °C, with sheath gas at 45 arbitrary units, auxiliary gas at 5 arbitrary units and the spray voltage at 4.0 kV. In positive/negative polarity switching mode, an m/z scan range from 70 to 850 was chosen and MS1 data was collected at a resolution of 70,000. The automatic gain control (AGC) target was set at 1 × 106 and the maximum injection time was 200 ms. The top 5 precursor ions were subsequently fragmented, in a data-dependent manner, using the higher energy collisional dissociation (HCD) cell set to 30% normalized collision energy in MS2 at a resolution power of 17,500. The sample volumes of 10 μl were injected. Data acquisition and analysis were carried out by Xcalibur 4.0 software and Tracefinder 2.1 software, respectively (both from Thermo Fisher Scientific).

### RNA sequencing

RNA was extracted using RNAeasy kit (Bio-Rad) to perform RNA-seq as previously described^22^. Libraries preparation, sequencing, and bioinformatics analysis of RNA sequencing were performed by Novogen (Novogen, Inc, Sacramento CA). Briefly, RNA integrity was assessed with Agilent Bioanalyzer 2100 to select RNA samples with RIN > 7.3–9.3. Two hundred fifty to 300 base pair insert cDNA libraries, non–strand-specific, were prepared with Next Ultra RNA Library Prep (New England Biolabs, Ipswich, MA) and sequenced with Illumina (San Diego, CA) HiSeq PE150 Platform ∼ 6G/sample Q30 > 90%. The reads were mapped to the human reference genome sequence using STAR v2.5 and v2.6.1, with a total mapping rate > 90% per sample. For gene expression level analysis and to calculate the fragments per kilobase of transcript per million mapped reads, HTSeq v0.6.1 was used. The differential expression analysis between two different groups was done with DESeq2 R package v2_1.6.3. The *P* values were adjusted using the Benjamini–Hochberg approach for controlling the false discovery rate, adjusted *P* < 0.05. TFCat and Cosmic databases were used to annotate the differential expressed gene. The enrichment analysis was done with cluster Profiler R package.

## Results

### Chronic Hepatic HKDC1 Overexpression in male mice results in impaired glucose tolerance but not in female mice

Our group recently observed that hepatic HKDC1 expression was elevated in NAFLD states in mouse models and human biopsy samples^10^. In addition, others have reported increased levels of HKDC1 in liver cancer, among other cancers^12,13,15,23^. Previously, we utilized adenovirus to acutely (1 week) overexpress HKDC1 in mice with specificity to the liver. There was a mild improvement in glucose homeostasis in this model, with no impairment in hepatic triglyceride nor glucagon levels, but the hepatocytes showed altered mitochondrial activity and dynamics when assessed ex vivo^10^. As metabolic alterations in homeostasis typically develops over a longer period such as seen in diseased states like NAFLD, we developed an approach to assess long-term effects of overexpression of hepatic HKDC1 in mice. Specifically, we injected AAV8-TBGp vectors to adult mice (age 10 weeks) and subsequently changed their standard diet to a low-fat diet. The mice achieved a long-term (16 weeks) stable hepatocyte-specific overexpression of human HKDC1 and this allowed us to evaluate if HKDC1 is sufficient to develop NAFLD-like characteristics compared to control mice (AAV-null) under non-stressed conditions.

**Figure 1:**
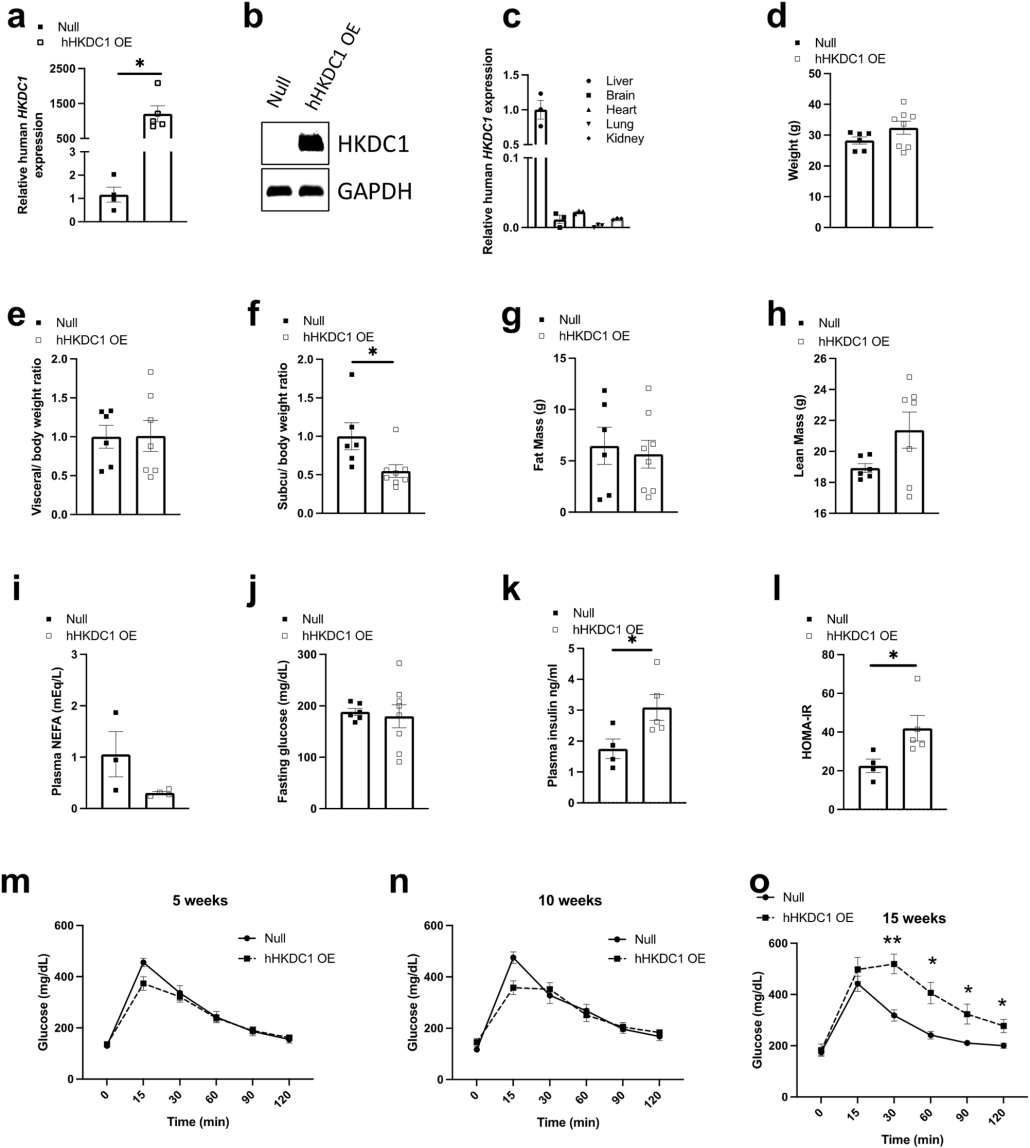
Chronic hepatic hHKDC1 overexpression results in impaired glucose tolerance in male mice. (**a**) mRNA expression of human HKDC1 in mouse livers relative to null, n = 4–5 mice/group. (**b**) Representative immunoblot of human HKDC1 in mouse liver, n = 6–8 mice/group. (**c**) mRNA expression of human HKDC1 in various mouse tissues relative to liver expression in hHKDC1 OE mice, n = 3 (**d**) Fasting body weights of male mice at 16 weeks, n = 6–8 mice/group. (**e**–**h**) Visceral and subcutaneous (subcu) fat weight relative to body weight, and also fat mass, and lean mass, respectively of male mice, n = 6–8 mice/group. (**i**) Plasma non-esterified fatty acids (NEFA) levels, n = 3–5 mice/group. (**j**) Fasting plasma glucose, (**k**) Fasting plasma insulin and (**l**) HOMA-IR, n = 4–8 mice/group. (**m**–**o**) Plasma glucose concentrations during an intraperitoneal glucose tolerance test (IPGTT) from mice at 5, 10 and 15 weeks post-injection, respectively, n = 6–8 mice/group. All values are means ± SEM. *p < 0.05, **p < 0.01 and *p < 0.05 (Unpaired Student t-test).

As seen in Fig. 1a, our AAV-HKDC1 (which will be referred to as hHKDC1 OE to signify human HKDC1 overexpression) approach resulted in stable overexpression of human *HKDC1* RNA in the liver with a concomitant increase in hepatic HKDC1 protein levels (Fig. 1b and Supplemental Fig. 1a) and not in other tissues (Fig. 1c). Our hHKDC1 OE mice did not have altered body weight (Fig. 1d, male and Supplemental Fig. 1b, female) or visceral fat (Fig. 1e, male and Supplemental Fig. 1c, female) after 16 weeks. However, subcutaneous fat was reduced in the hHKDC1 OE mice as compared to the control based on dissection (Fig. 1f, male and Supplemental Fig. 1d, female), while overall fat mass (Fig. 1g, male and Supplemental Fig. 1e, female) and lean mass (Fig. 1h, male and Supplemental Fig. 1f, female) were not significantly different. Furthermore, to assess whether this reduction in subcutaneous fat was caused by an increase in lipolysis, we measured non-esterified fatty acid (NEFA) levels in our male mice, however, a mild non-significant reduction was observed (Fig. 1i). To further investigate the cause of this reduction, we performed RNA sequencing analysis on these fat depots in our male mice and found adipocyte apoptosis and inflammatory pathways were up-regulated (Supplemental Fig. 2a), while various pathways involving fat metabolism and transport were down-regulated (Supplemental Fig. 2b). In addition, we interestingly found that the genes involved in white adipose browning such as uncoupling protein 1 (*Ucp1*), iodothyronine deiodinase 2 (*Dio2*), and cell death activator (*Cidea*) were increased while genes involved in adipogenesis were decreased (Supplemental Fig. 2c). Thus, chronic hepatic overexpression of HKDC1 did not significantly alter body weight or composition, however, did affect subcutaneous fat depots with gene alterations in inflammatory, fat metabolism, browning, and adipogenesis pathways.

As liver is a central organ in glucose homeostasis, we next assessed characteristics of whole body glucose homeostasis after 16 weeks. There were no changes noted in fasting glucose for male mice after 16 weeks (Fig. 1j) but had increased fasting insulin levels and HOMA-IR (Fig. 1k and l, respectively), while the fasting glucose levels were significantly reduced in female mice (Supplemental Fig. 3a) with no change in fasting insulin and HOMA-IR (Supplemental Fig. 3b and c, respectively). Moreover, while our male mice did not exhibit changes in glucose tolerance after an IPGTT at 5 and 10 weeks (Fig. 1m and n), there was significant impairment noted at 15 weeks (Fig. 1o). In contrast, we did not note changes to glucose tolerance at 5, 10, or 15 weeks in our female mice (Supplemental Fig. 3d, e, f, respectively).

### Chronic hepatic HKDC1 overexpression results in hepatomegaly in male mice

**Figure 2:**
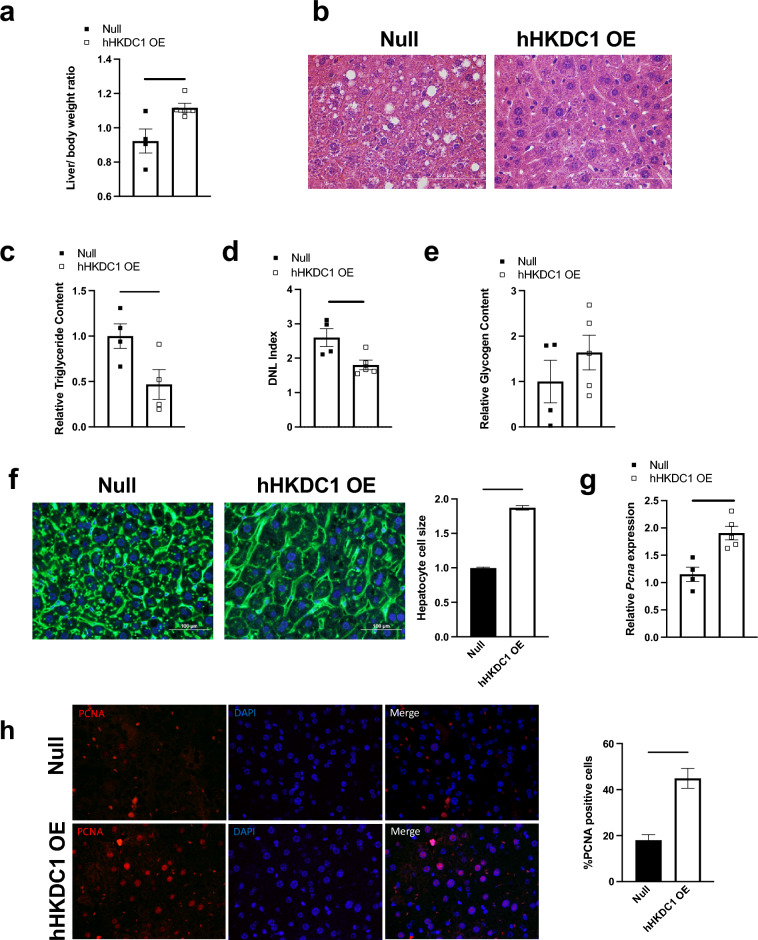
Chronic hepatic hHKDC1 overexpression results in hepatomegaly in male mice. (**a**) Liver weight normalized to body weight ratio, n = 4–5 mice/group. (**b**) Representative liver sections stained with hematoxylin–eosin (H&E). (**c**) Hepatic triglyceride content, (**d**) Hepatic de novo lipogenesis (DNL) index, (**e**) Hepatic glycogen content, n = 4–5 mice/group for (**c**–**e**). (**f**) Immunofluorescence microscopy of liver Wheat Germ Agglutinin staining (WGA); Green: WGA, Blue: DAPI and corresponding liver cross-sectional area measurements, n = 3 mice/group. (**g**) mRNA expression of liver proliferating cell nuclear antigen (PCNA) relative to null, n = 4–5 mice/group. (**h**) Immunofluorescence microscopy of liver PCNA and corresponding quantification; Red: PCNA, Blue: DAPI, and number of PCNA detected per field, n = 3 mice/group. All values are means ± SEM. *p < 0.05, **p < 0.01 and ****p < 0.0001 (Student t-test).

In addition to lower subcutaneous fat, the hHKDC1 OE male mice had elevated liver weights compared to the control after 16 weeks (Fig. 2a), while the female mice liver weights were unchanged (Supplemental Fig. 3g). Given that the female mice did not exhibit changes in glucose tolerance nor changes in liver size, we continued our studies focusing only on the hHKDC1 OE male mice. To investigate the cause of increased liver mass, we first performed a hematoxylin and eosin (H&E) stain to gain an understanding of the hepatocyte morphology (Fig. 2b). Interestingly, we noted enlarged nuclei and hepatocyte size but a decrease in fatty vacuoles. To verify there is a reduction in lipids, we measured liver triglycerides and observed a significant reduction in the hHKDC1 OE mice (Fig. 2c) While there are various reasons why triglyceride levels could be decreased, we found that hepatic lipid synthesis, as measured by the de novo lipogenesis (DNL) index, was also significantly decreased (Fig. 2d). Moreover, we also measured glycogen storage levels, however, observed only a mild, non-significant increase (Fig. 2e). Fat nor glycogen storage explained the enlarged liver phenotype, however, we did note that hepatocytes from the hHKDC1 OE mice appeared enlarged. We further investigated this finding with immunofluorescence using WGA staining and calculation of size and noted significantly increased size (Fig. 2f). Next, we proceeded to investigate whether the hepatocytes possessed proliferation potential as a proxy for active proliferation. We first measured PCNA (a marker of active proliferating cells) RNA expression and observed a significant increase (Fig. 2g), as well as, increase co-localization of PCNA with DAPI (nuclear stain), compared to the control mice (Fig. 2h). To assess if there were signs of liver injury that could explain the response to hepatocyte cell growth, we measured plasma ALT and AST levels. However, there were undetectable or low levels in our controls and experimental groups and they were not significantly different between groups (data not shown). While hepatomegaly is often caused by fat and/or glycogen accumulation, our HKDC1 OE mice had enlarged liver predominantly related to an increase in cell size and proliferation capacity.

### Chronic hepatic overexpression of HKDC1 up-regulates the transcription factor YAP

**Figure 3:**
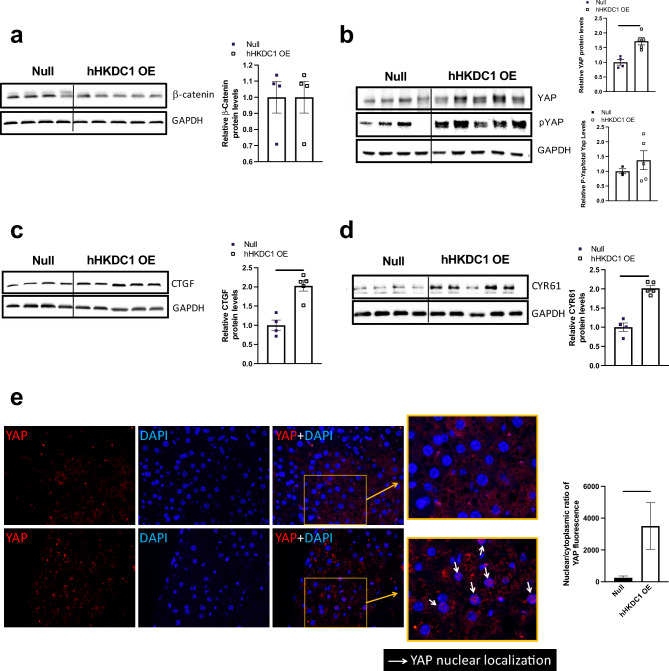
Chronic hepatic overexpression of hHKDC1 up-regulates the transcription factor YAP. (**a**) Immunoblot of Beta-catenin normalized to GAPDH and corresponding quantification. (**b**) Immunoblot of yes-associated protein (YAP) normalized to GAPDH and corresponding quantification, immunoblot of phosphorylated YAP at serine 127 (pYAP) normalized to GAPDH and corresponding quantification of the ratio between pYAP to total YAP. (**c**) Immunoblot of connective tissue growth factor (CTGF) normalized to GAPDH and corresponding quantification. (**d**) Immunoblot of cysteine-rich angiogenic inducer 61 (CYR61) normalized to GAPDH and corresponding quantification. n = 4–5 mice/group for (**a**–**d**). (**e**) Immunofluorescence microscopy of liver sections; Red: YAP, Blue: DAPI, n = 3 mice/group. All values are means ± SEM. *p < 0.05, **p < 0.01, ****p < 0.0001 (Student t-test).

We next wanted to assess key pathways known to be involved in liver size regulation. A study from 2016 demonstrated that the silencing of HKDC1 decreased proliferation and migration via the downregulation of the β-catenin and C-Myc pathways in hepatocellular cell lines^15^. Thus, we first proceeded to assess if chronic overexpression of hepatic HKDC1 affected whole cell β-catenin levels (Fig. 3a) and its sub-cellular localization levels (Supplemental Fig. 4), however, we did not note any differences with our chronic overexpression mouse model. Of note, another recent study by Bisso et al. found that conditional overexpression of Myc and β-catenin in hepatocytes increased proliferation specifically via the transcription factors Yap/Taz and their downstream targets^24^. Considering this, we examined the protein levels of YAP and found that our hHKDC1 OE mice had significantly elevated levels of YAP compared with null mice. Next, we examined the phosphorylation of YAP at serine 127 (which indicates sequestration in the cytosol), which when normalized to total YAP levels was not significantly increased (Fig. 3b), indicating possibly more nuclear YAP. We then investigated whether YAP’s downstream targets were affected and found that connective tissue growth factor (CTGF) (Fig. 3c) and cysteine-rich angiogenic inducer 61 CYR61 (Fig. 3d), were significantly elevated. To further verify higher localization of YAP to the nucleus, we co-stained YAP and DAPI and identified co-localization was significantly elevated in the hHKDC1 OE liver sections (Fig. 3e). Taken together, hepatic HKDC1 overexpression is found to correlate with the upregulation of YAP, its localization to the nucleus, and its downstream targets.

### Up-regulation of YAP, cell size, and proliferative capacity is moderately dependent on HKDC1 expression

**Figure 4:**
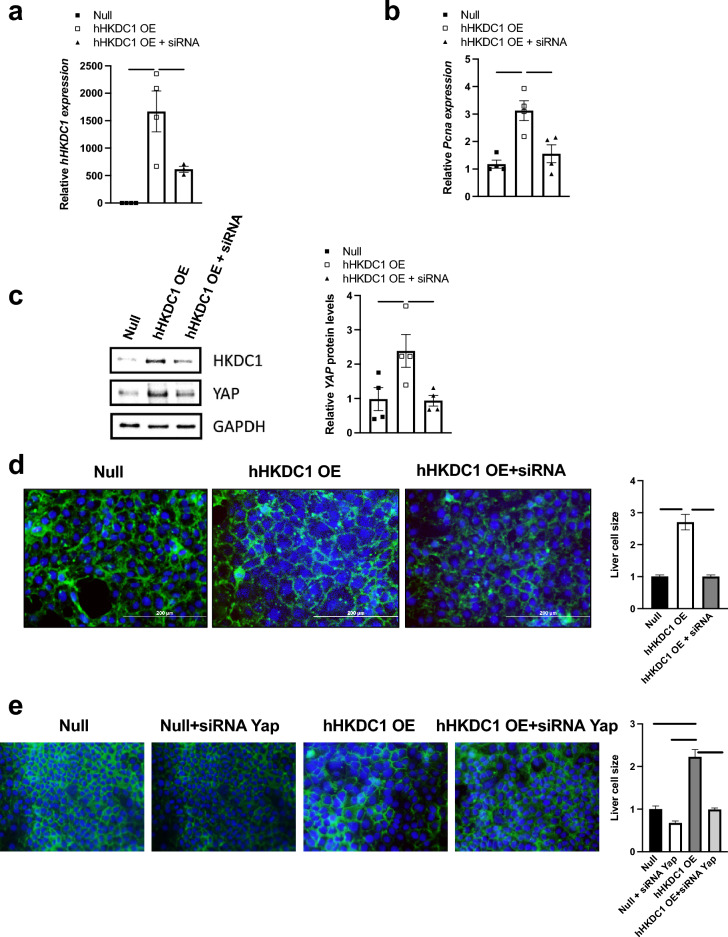
Up-regulation of YAP, cell size, and proliferative capacity is moderately dependent on HKDC1 expression. (**a**) mRNA expression of hHKDC1 and (**b**) PCNA in the stable OE of hHKDC1 in AML-12 cells and stable OE of hHKDC1 with siRNA against HKDC1 normalized to β-actin relative to null, n = 3–4. (**c**) Immunoblots of YAP normalized to GAPDH and corresponding quantification. (**d**) Immunofluorescence microscopy of WGA stain of stable OE of null AML-12 cells, stable OE of hHKDC1, and stable OE of hHKDC1 with siRNA against HKDC1 and corresponding size quantification, n = 3, > 50 cells each. (**e**) Immunofluorescence microscopy of WGA stain of null AML-12 cells, cells with siRNA against Yap, stable OE of hHKDC1, and stable OE of hHKDC1 with siRNA against Yap and corresponding size quantification; n = 3, > 25 cells each; Green: WGA, Blue: DAPI and corresponding area of measurements. All values are means ± SEM. *p < 0.05, **p < 0.01, ****p < 0.0001 (One-way ANOVA).

To assess whether YAP and its role in proliferation is influenced by HKDC1, we first began by establishing a stably-overexpressing hHKDC1 (will also be referred to as hHKDC1 OE) in a mouse hepatocyte line, AML-12, and verified its overexpression and specificity of siRNA knockdown (Fig. 4a). We observed that hHKDC1 OE increased *Pcna* RNA expression, similar to what we observed in vivo, and that silencing of HKDC1 significantly reduced *Pcna* back to baseline (Fig. 4b). We also noted an increase in YAP protein levels in the hHKDC1 OE cells and with silencing of HKDC1 decreasing YAP protein comparable to baseline (Fig. 4c). Next, we verified that the observed increase in hepatocyte size was due to HKDC1 expression via WGA staining and furthermore showed that with silencing of HKDC1, the hepatocytes reverted to a size comparable to Null (Fig. 4d). To then determine whether the increase in hepatocyte size is Yap-dependent, we assessed the hepatocyte cell size difference between the hHKDC1 OE cells and hHKDC1 OE with siRNA of *Yap* and noted a significant increase in hepatocyte size with the hHKDC1 OE cells and found that silencing *Yap* decreased the cell size (Fig. 4e). Overall, HKDC1 expression induces an increase in YAP and leads to an increase in cell size that is at least, in part, Yap-dependent.

### Chronic hepatic overexpression of HKDC1 does not impact other HK expression but causes an increase in HK activity and decreases PKM2 and lactate

**Figure 5:**
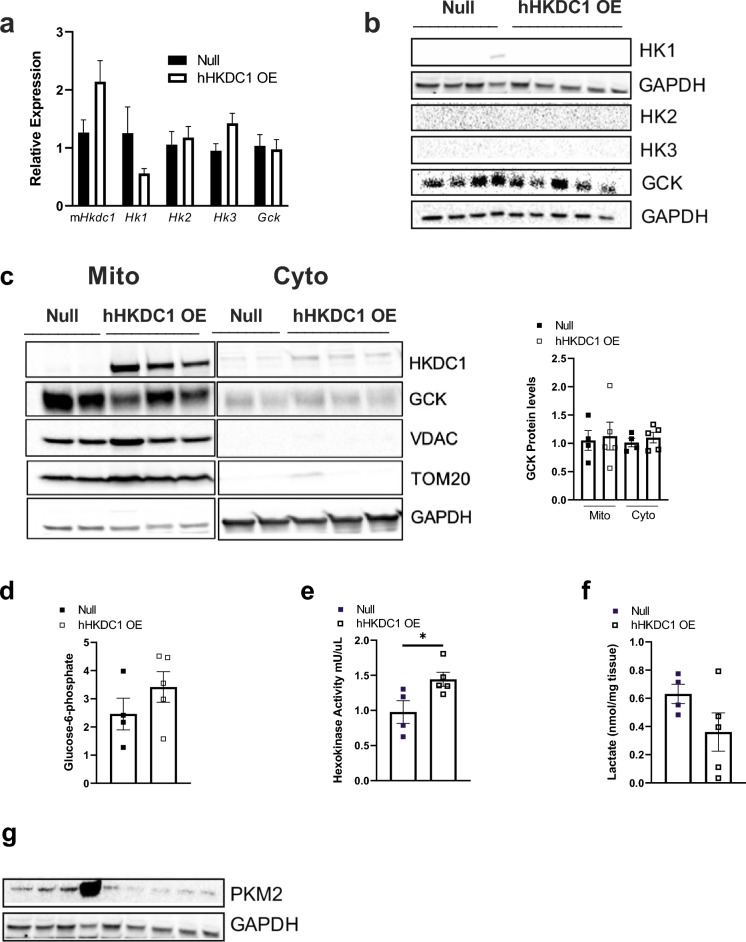
Chronic hepatic overexpression of HKDC1 does not impact other hexokinases but causes an increase in HK activity and decreases in PKM2 and lactate. (**a**) mRNA expression of mouse Hkdc1, Hk1, Hk2, Hk3 and Gck in hHKDC1 OE and null mouse livers relative to null, n = 4–5 mice/group. (**b**) Immunoblots of hexokinase 1 (HK1), hexokinase 2 (HK2), hexokinase 3 (HK3) and glucokinase (Gck) normalized to corresponding GAPDH of hHKDC1 OE and null mouse livers. (**c**) Immunoblots of HKDC1, GCK, GAPDH (cytosolic housekeeping), and translocase of the outer mitochondrial membrane complex subunit 20 (TOM20, mitochondrial housekeeping) on mitochondrial (mito) and cytosolic (cyto) fractions of mouse livers and corresponding quantification of GCK. (**d**) Glucose-6-phosphate levels, (**e**) Hexokinase activity and (**f**) Lactate levels in mouse liver tissue, n = 4–5 mice/group. (**g**) Immunoblots of PKM2 and GAPDH. All values are means ± SEM. *P < 0.05 (Student t-test).

To elucidate whether chronic HKDC1 expression influences changes in expression of other HK isoforms, we first measured HK hepatic expression and protein levels in our chronic hepatic hHKDC1 OE mouse model. We observed no significant differences in RNA expression (Fig. 5a), no detectable HK protein levels of HK1, HK2, or HK3, and no changes to the dominant hepatic hexokinase isoform, GCK (Fig. 5b). Given that GCK’s sub-cellular localization can direct downstream cellular metabolism and the metabolic fate of the cell^25,26^, we assessed whether overexpression of HKDC1 affected GCK localization. As we noted previously^10^, HKDC1 localizes largely to the mitochondria upon overexpression, and here, we observed no major alterations in GCK localization (Fig. 5c). Next, because we observed chronic HKDC1 increases hepatocyte size and proliferation capacity and that there is a high association of HKDC1 with liver cancer^22^ and other cancers, we examined whether HKDC1 contributes to specific aspects of the Warburg-effect. This phenomenon switches metabolism to take up more glucose and preferentially produces lactate, even in the presence of oxygen and functional mitochondria^27^. While not significant, we noted a trending increase in glucose-6-phosphate levels (Fig. 5d), which may be an indication of increased glucose uptake. However, we also noted significantly increased overall cellular hexokinase activity (Fig. 5e), which could also have also contributed to the observed increased glucose-6-phosphate levels. Interestingly, while not significant, lactate levels down-trended in the hHKDC1 OE mice (Fig. 5f) in addition to pyruvate kinase M2 (PKM2) levels (Fig. 5g), an enzyme that helps give rise to the Warburg effect^28^. Overall, chronic hHKDC1 did not alter HK levels or localization and while HKDC1 contributes to increased hepatocyte cell growth, it did not contribute to the canonical signs defined by the Warburg effect.

### Chronic hepatic overexpression of HKDC1 shifts metabolism towards anabolism

**Figure 6:**
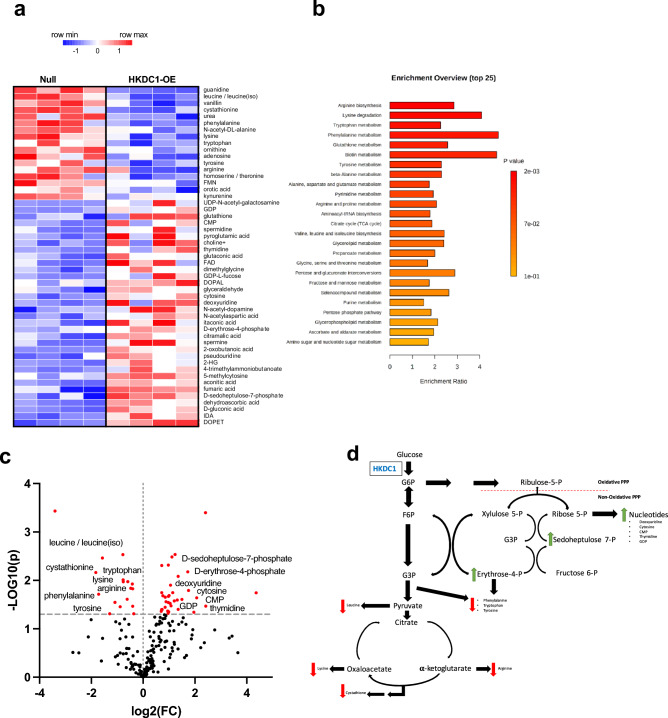
Chronic hepatic overexpression of HKDC1 shifts metabolism towards an anabolism. (**a**) Heat map of steady-state metabolomics performed in mouse livers normalized to total-ion-content. (**b**) Metabolite enrichment analysis of the top 25 metabolic pathways. (**c**) Important features selected by volcano plot with fold change (x-axis), t-tests threshold (y-axis) 0.05. Red circles represent above threshold. (**d**) Summary of steady state metabolites. Green arrows: Increase with HKDC1 OE, Red arrows: Decrease with HKDC1 OE. Both fold changes and *p* values are log transformed. All values are means ± SEM. *p < 0.05 (Student t-test). n = 4–5 mice/group.

We lastly explored how chronic HKDC1 overexpression alters downstream metabolism that might explain the hepatocyte cell growth phenotype via untargeted metabolomics of the mouse livers. The steady state metabolomics was performed on the liver extracts of mice after a 4 h fast. The metabolites showed distinct hepatic metabolic profiles with 51 metabolites significantly different between the groups (Fig. 6a). Examining the enrichment pathway analysis (Fig. 6b), we observed that biosynthesis/degradation pathways of multiple amino acids are impacted, as well as TCA, pentose phosphate pathway, purine and pyrimidine pathways were influenced. Multiple amino acids were decreased in HKDC1 OE liver samples, including tyrosine, tryptophan, phenylalanine, leucine, lysine, and arginine but nucleotides such as deoxyuridine, cytosine, CMP, thymidine and GDP and their precursors from the pentose phosphate pathway intermediates D-sedoheptulose-7-phosphate and D-erythrose-4-phosphate were increased compared to the control samples (Fig. 6c). Overall, chronic overexpression of hepatic HKDC1 shifts metabolites away from catabolic pathways (TCA cycle) and towards anabolic pathways such as the pentose phosphate pathway (Fig. 6d).

## Discussion

We have previously examined HKDC1’s role in the liver following acute (seven days) adenoviral overexpression of HKDC1^14^. While we did not note changes in energy storage within the liver, we did discover a mild improvement of glucose intolerance during an IPGTT^10^. Similar to this report, we have found that after 16 weeks of AAV-8 targeted hepatic chronic overexpression of human HKDC1, there were also no major changes in energy storage. There were no changes in glucose tolerance at the five and 10-week time points but, interestingly, there was a significant impairment noted at 15 weeks in our chronic overexpression male mice. These data suggest acute versus chronic hepatic overexpression of HKDC1 differentially impacts whole glucose body tolerance. In addition, we observed higher fasting insulin levels in the male mice, further suggesting the mice were developing insulin resistance. While we originally expected there to be an increase in energy storage given the higher levels of insulin, we instead noted a significant increase in liver mass and hepatocyte growth, which undoubtedly requires an increase in glucose energy demands. As evidenced during times of accelerated hepatocyte growth, such as after liver resections or HCC, studies have described that higher insulin levels can result in the development of insulin resistance^29,30^. We have also shown evidence that glucose-6-phosphate preferentially shuttles through the PPP resulting in increased anabolic metabolites leading to increased nucleotide synthesis and decreased catabolic metabolites, which may be contributing to the hepatocyte’s cell growth and division potential.

Our female mice, on the other hand, did not have increased insulin nor enlarged livers, which suggests there is perhaps a sex-specific protection against the development of insulin resistance. Notably, female mice are largely protected from diet-induced obesity, due to sex-differences^31^. Also, in humans, sex differences exist with females having lower incidence of NAFLD and HCC^32^. While these sex differences are well described, it is also interesting given HKDC1 was first discovered in genetic studies focused on gestational glucose metabolism^7^. While we did not observe some changes in female mice as we did in male mice, we did observe the reduction in the subcutaneous fat in both sexes and addressing the role of HKDC1 in sex differences in these metabolic outcomes will be an important future direction.

As noted above, we observed the interesting finding of reduced subcutaneous fat depots. Therefore, to gain an understanding of the subcutaneous fat processes that were occurring in response to chronic hepatic hHKDC1, we performed RNA sequencing on the subcutaneous adipose tissues of the male mice. The main pathways that were enhanced were inflammatory and apoptosis processes, as well as, genes involved with white adipose tissue browning such as Ucp1, Cidea, and Dio2. The increase in insulin resistance noted in our male mice may go in line with the notion that elevated insulin levels causes activation of adipose tissue inflammation and apoptosis^33–35^. Furthermore, the genes involved in the browning of white adipose tissue may also help support the finding of reduced subcutaneous mass due to an increase in energy expenditure rather than an increase in lipolysis as noted by the mild to no change in NEFA levels nor lipolysis genes. Given the influence hepatic HKDC1 expression has on adipose tissue, future studies will examine the metabolic crosstalk processes between the liver and subcutaneous fat and potential mechanisms of energy expenditure by evaluating whether there are changes in brown adipose tissue mass and body temperature.

A study by Zhang et al.^15^ found that in hepatocarcinoma cell lines, silencing of HKDC1 caused an inhibition of cell proliferation and migration in part due to the suppression of the Wnt/β-catenin pathway. Thus, we began by exploring whether chronic overexpression of HKDC1 was sufficient to influence β-catenin protein levels. After observing no changes in β-catenin levels nor nuclear localization, we considered the possibility that the effect on β-catenin noted in that study was due to acute silencing of HKDC1 and therefore, we explored its downstream pathways that could be affected related to hepatocyte proliferation. It was reported that when Myc and β-catenin were co-expressed in the livers of mice, a strong cooperative effect on liver tumorigenesis was found to be mediated by YAP/Taz^24^. Other studies have elucidated YAP’s role as a transcription factor for the induction of genes involved in cell proliferation and inhibition of apoptosis. Furthermore, Su et al. reported that YAP activation is insufficient to promote cellular proliferation in normal livers but rather YAP-expressing hepatocytes proliferate only in the presence of hepatocyte damage or inflammation^36^. It is difficult to discern why we did not observe detectable markers of hepatocyte injury (ALT and AST) and yet, our overexpression of hHKDC1 model still resulted in significantly elevated YAP protein levels and increased localization into the nucleus, where it is well known to serve as a transcription factor. Perhaps given the chronic nature of HKDC1 overexpression, the ALT and AST levels may have been higher at more acute time points and leveled out after 16 weeks. It is also important to consider that up to 50% of NAFLD patients can have normal ALT and AST levels, and therefore, the normal ALT and AST levels does not rule out that there are underlying inflammatory or liver injury processes occurring^37^. In addition, while there appeared to be an increase in the phosphorylation of YAP at serine 127, a consequence of which sequesters YAP in the cytoplasm and limits its transcriptional function^38^, when the phosphorylation of YAP is normalized to the total levels, there is not actually a rise in phosphorylation when compared to the null. Furthermore, we verified that YAP preferentially localized to the nucleus rather than the cytosol and increased its main targets CTGF and CYR61, both of which are involved in hepatocyte proliferation in response to injury and have roles in HCC progression^39^. Moreover, our in vitro studies with the hHKDC1 OE in AML-12 cells similarly showed increased in YAP levels with subsequent cell growth and further highlighted how changes in YAP expression is modulated by HKDC1 expression.

The question of whether increased YAP signaling plays a role in the metabolic changes that leads to the insulin resistance phenotype noted in our mouse model remains unclear. A study by Sayedyahossein et al.^40^ suggests that insulin signaling causes an increase in YAP phosphorylation and therefore significant reduction of YAP and its activity in the nuclei of liver cell lines. Given that we see increased insulin levels with increased activation of YAP, this could suggest that the activation of YAP is the initiating pathway that may be causing insulin resistance and not the other way around. This also aligns with our in vitro finding that YAP expression and nuclear localization is at least, in part, dependent on its upstream modulator, HKDC1. Additionally, others have observed that YAP deletion or phosphorylation at serine 127 improves insulin resistance^41,42^. While these associations suggest a role for hepatic Hippo/YAP signaling in insulin resistance, future studies need to explore the causal link between HKDC1 and YAP signaling and whether the changes in whole body glucose homeostasis is dependent or independent of YAP signaling.

In our previous paper, HKDC1 in hepatocytes largely localized to the mitochondria and caused signs of mitochondrial dysfunction^14^. Additionally, we found protein purified HKDC1 to contribute low-affinity hexokinase activity and similarly, when we generated a stable HKDC1 overexpression cell line in MI5-4 CHO cells (an established line with minimal HK activity), we observed HKDC1 produced a modest amount of total HK activity at all glucose concentrations tested^10^. Given that glucokinase is the main HK in normal liver metabolism, we first examined whether chronic overexpression of HKDC1 affected GCK localization since it has been found to localize to the outer mitochondrial membrane^43^. However, we did not note any significant changes and proceeded to examine whether there were any differences in hexokinase activity. We did find there to be significantly increased overall cellular hexokinase activity and mildly increased levels of glucose-6-phosphate but decreased lactate, which led us to examine how this increased HK activity could be affecting downstream metabolism. Consistent with the findings of increased hepatocyte size and proliferation, the pathways that were upregulated were related to amino acid anabolism and nucleotide synthesis. While the increase in HK activity and cell growth supports a Warburg-effect picture, the trending decrease in lactate, no detectable levels of HK2 (the hexokinase that predominates in liver cancer) and decreased PKM2 levels suggests that the downstream metabolism changes that HKDC1 influences may differ from that of HK2. However, a limitation in our mouse model is that our chronic overexpression of HKDC1 stopped at 16 weeks and is perhaps was not enough time to produce a full metabolic switch towards cancer metabolism.

Given that our lab and others have noted that in both mouse and human livers HKDC1 expression increases in stressed states such as NAFLD, alcoholic steatosis, and HCC and that studies have found increased activity of YAP in early liver cancer development^44^, it is plausible that HKDC1’s role in liver pathologies such as NAFLD and HCC tumorigenesis may, in part, be mediated via its relationship to YAP signaling. In sum, these data established that chronic HKDC1 expression impacts whole body glucose homeostasis, liver size and hepatocyte proliferation capacity. Next steps will be to address the role of HKDC1 over-expression or deletion in pathophysiological states such as NAFLD or HCC.

## Supplementary Information

Supplementary Information.

## Data Availability

RNA sequences data was deposited in NCBI. The accession number is GSE227535. Link to data: https://www.ncbi.nlm.nih.gov/geo/query/acc.cgi?acc=GSE227535.
