# Supplementary material for: Liver-specific overexpression of HKDC1 increases hepatocyte size and proliferative capacity
Source: Sci Rep. 2023 May 17;13:8034. doi: 10.1038/s41598-023-33924-3 (PMC10192376; doi:10.1038/s41598-023-33924-3)
Supplement: Supplementary file 1 — Supplementary Information. [file 41598_2023_33924_MOESM1_ESM.docx]

**Liver-Specific Overexpression of HKDC1 Increases Hepatocyte Size and Proliferative Capacity**

Carolina M Pusec^1^, Vladimir Ilievski^1^, Adam De Jesus^2^, Zeenat Farooq^1^, Joseph L Zapater^1,3^, Nadia Sweis^1^, Hagar Ismail^1^, Md Wasim Khan^1^, Hossein Ardehali^2^, Jose Cordoba-Chacon^1^, Brian T. Layden^1,3^*

^1^Division of Endocrinology, Diabetes, and Metabolism, Department of Medicine, University of Illinois at Chicago, Chicago, Illinois.

^2^Northwestern University Feinberg School of Medicine, Chicago, Illinois.

^3^Jesse Brown VA medical center, Chicago, Illinois.

^*^Address all correspondence to Brian T. Layden, blayde1@uic.edu

Supplemental material

**Female overexpression verification and weight analysis.** (a) Immunoblot of human HKDC1 in female mouse liver. (b) Fasting body weights of female mice at 16 weeks, n=9-12. (c-f) Visceral and subcutaneous (subcu) fat weight relative to body weight, and also fat mass, and lean mass, respectively of female mice, n=4-12 mice/group. All values are means ± SEM. ****p<0.0001 (Student t-test).

**RNA sequencing analysis of subcutaneous fat depots.** PANTHER Gene Ontology analysis of (a) Up-regulated genes and (b) Down-regulated genes comparing hHKDC1 OE mouse subcutaneous fat with null; genes above threshold of 1.2 log fold change were included (1658 genes for up-regulated and 488 for down-regulated). (c) Metabolic processes and corresponding representative genes involved in subcutaneous fat including browning, lipolysis and synthesis, mitochondrial oxidative phosphorylation (Mito OxPhos), fatty acid (FA) and glucose transporter, and adipogenesis. Fold change is relative to null, n=3 mice/group. The p values were adjusted using the Benjamini-Hochberg approach, adjusted p<.05. The enrichment analysis was done with cluster Profiler R package. *p<0.05, **p<0.01, ****p<0.0001.

**Female glucose homeostasis characterization.** (a) Fasting plasma glucose (b) Fasting plasma insulin and (c) HOMA-IR, n=9-12 mice/group. (d-f) Plasma glucose concentrations during an IPGTT from mice 5, 10 and 15 weeks post-injection, respectively. All values are means ± SEM. ***P<0.001 (Student t-test).


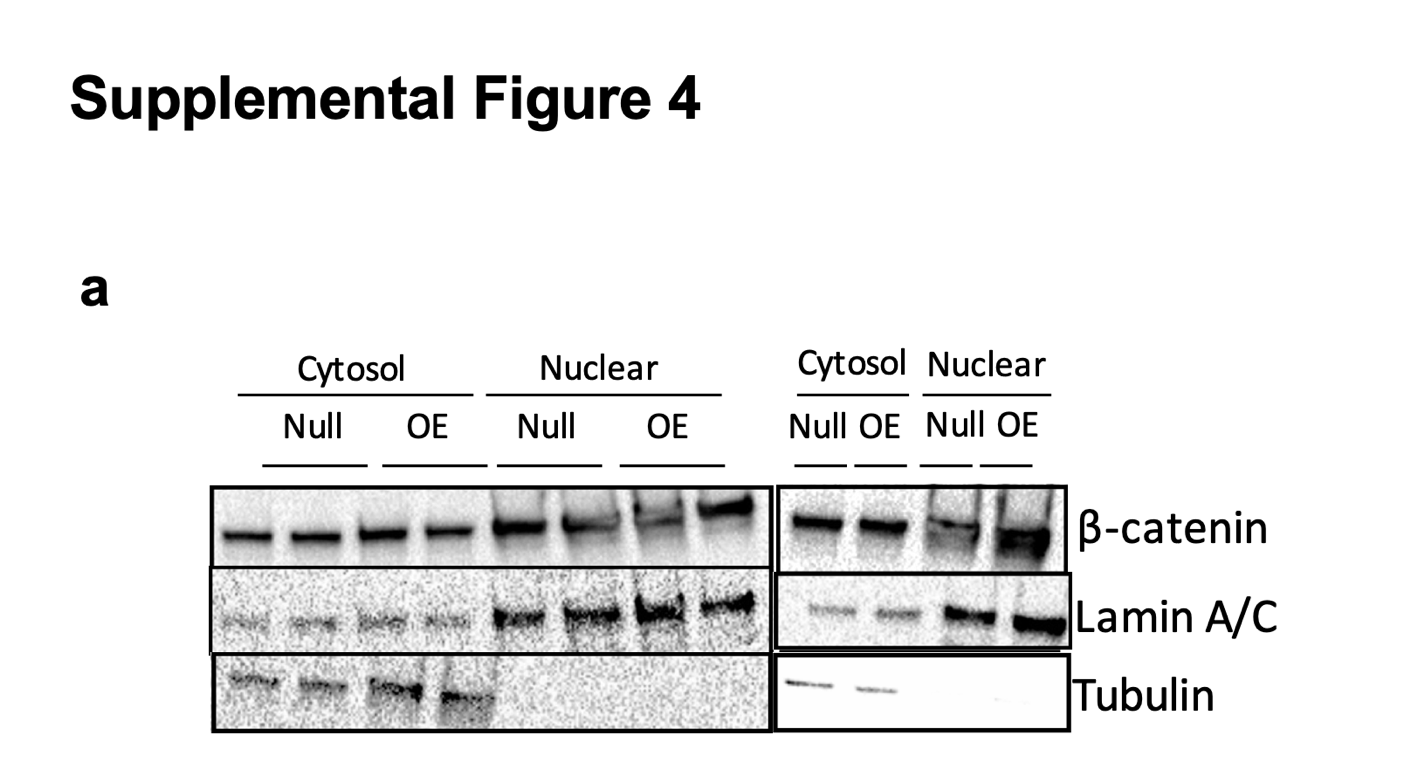


**Beta-catenin sub-cellular localization with hHKDC1 overexpression in liver.** Immunoblots of β-catenin, GAPDH (cytosolic housekeeping), and Lamin A/C (nuclear housekeeping) on cytosolic and nuclear fractions of hHKDC1 OE and null mouse livers. n=3 male mice/group.

**Original Western Blot Images Under Multiple Exposures**

**Supplemental Figure 5**

**Cropped blot in the main manuscript**

**Figure 1b**

Lanes 1, 2 = Null

Lanes 3, 4, 5 = hHKDC1 OE

**
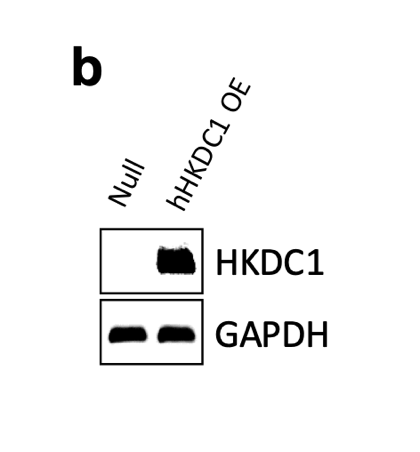
**

**Original uncropped blots**

**Figures 3a-d**

Lanes 1-4 = Null

Lanes 5-9 = hHKDC1 OE

Lanes 10-14 = Not shown in paper

**Cropped blots in the main manuscript**


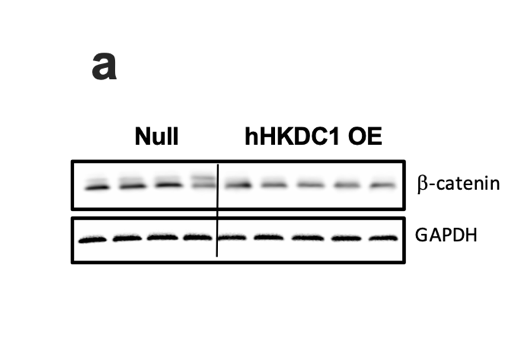

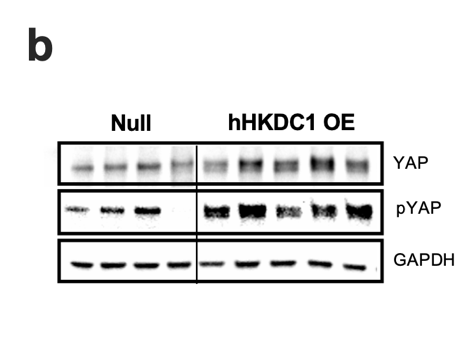


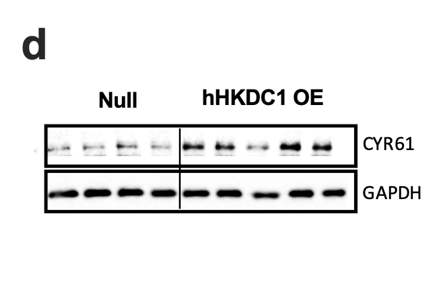

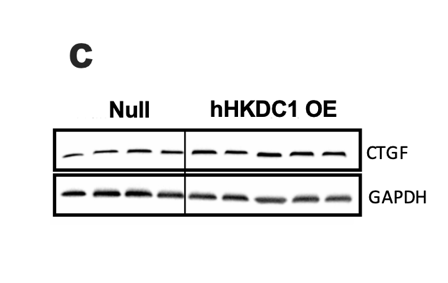


**Original uncropped blots**

**a**

**b**

**c**

**d**

**Figure 4c**

**Cropped blots in the main manuscript**


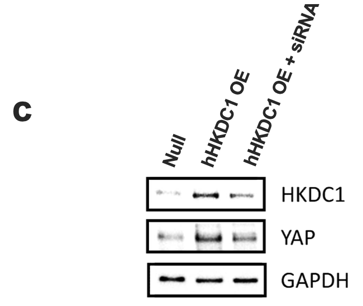


hHKDC1 OE+siRNA

hHKDC1 OE+siRNA

hHKDC1 OE

hHKDC1 OE

Null

Null

Set #2

Set #1

**Original uncropped blots**

**Cropped blots in the main manuscript**

**Figure 5b**

Lanes 1-4 = Null

Lanes 5-9 = hHKDC1 OE


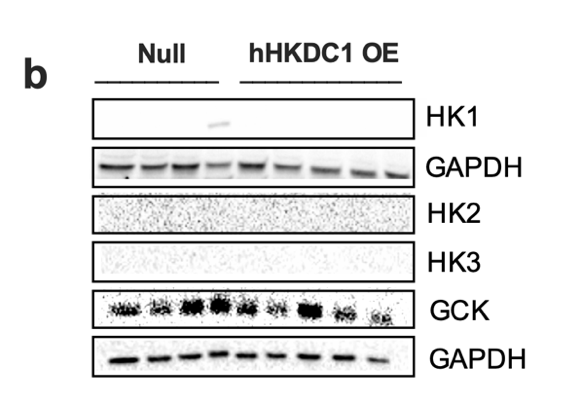


**Original uncropped blots**

**Figure 5c**

**Cropped blots in the main manuscript**


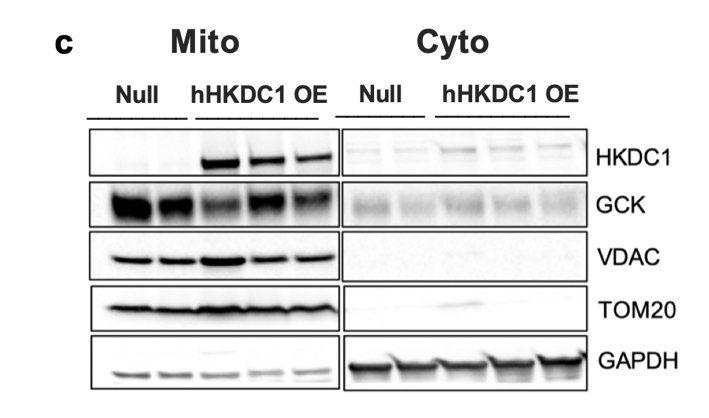


**Original uncropped blots**

7.2 sec

4 sec

4 sec

2 sec

**Figure 5g**

**Cropped blots in the main manuscript**


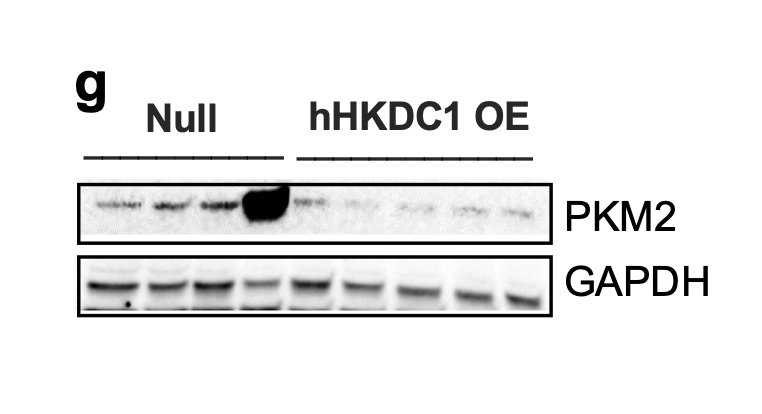


**Original uncropped blots**

**Supplemental Figure 1a**

**Cropped blots**

Female mice

Lanes 1-7 =Null

Lanes 8-14 = hHKDC1 OE


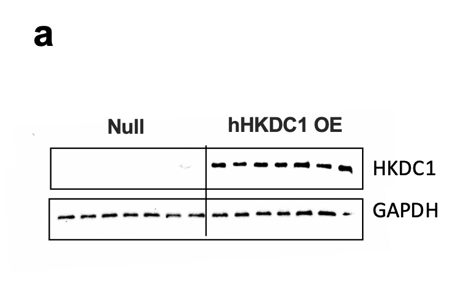


**Original uncropped blots**

**Supplemental Figure 4a**

I set

1, 2 Null; 3, 4 OE; 5, 6 Null; 7, 8 OE

_____________ _____________

Cytoplasm Nuclear

II set

1 Null; 2 OE; 3 Null; 4 OE _____________ _____________

Cytoplasm Nuclear

**
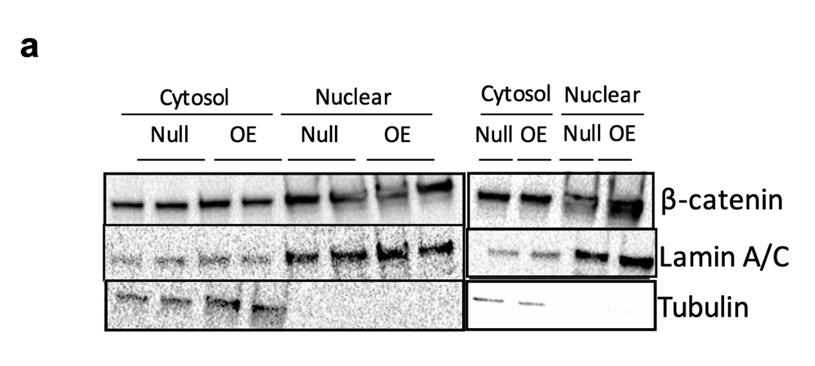
**

**Cropped blots**

**I set**

**II set**

**Original uncropped blots**

β-catenin

I set

β-catenin

II set

I set

Lamin A/C

Lamin A/C

II set

I set

I set

Tubulin

II set
